# Glycolysis and the Pentose Phosphate Pathway Promote LPS-Induced NOX2 Oxidase- and IFN-β-Dependent Inflammation in Macrophages

**DOI:** 10.3390/antiox11081488

**Published:** 2022-07-29

**Authors:** Jonathan R. Erlich, Eunice E. To, Raymond Luong, Felicia Liong, Stella Liong, Osezua Oseghale, Mark A. Miles, Steven Bozinovski, Robert D. Brooks, Ross Vlahos, Stanley Chan, John J. O’Leary, Doug A. Brooks, Stavros Selemidis

**Affiliations:** 1 School of Health and Biomedical Sciences, RMIT University, Bundoora 3083, Australia; jonathan.erlich@rmit.edu.au (J.R.E.); eunice.e.to@gmail.com (E.E.T.); felicia.liong@rmit.edu.au (F.L.); stella.liong@rmit.edu.au (S.L.); osezua.oseghale@hudson.org.au (O.O.); mark.miles@rmit.edu.au (M.A.M.); steven.bozinovski@rmit.edu.au (S.B.); ross.vlahos@rmit.edu.au (R.V.); stanley.chan@rmit.edu.au (S.C.); 2 F.M. Kirby Neurobiology Centre, Boston Children’s Hospital, Harvard Medical School, Boston, MA 02115, USA; 3 Department of Pharmacology, Biomedicine Discovery Institute, Monash University, Clayton 3800, Australia; raymond.luong91@gmail.com; 4 Clinical and Health Sciences, University of South Australia, Adelaide 5001, Australia; robert.brooks@unisa.edu.au (R.D.B.); doug.brooks@unisa.edu.au (D.A.B.); 5 Discipline of Histopathology, School of Medicine, Trinity Translational Medicine Institute (TTMI), Trinity College Dublin, D08 XW7X Dublin, Ireland; olearyjj@tcd.ie; 6 Sir Patrick Dun’s Laboratory, Central Pathology Laboratory, St James’s Hospital, D08 XW7X Dublin, Ireland; 7 Emer Casey Research Laboratory, Molecular Pathology Laboratory, The Coombe Women and Infants University Hospital, D08 XW7X Dublin, Ireland; 8 CERVIVA Research Consortium, Trinity College Dublin, D08 XW7X Dublin, Ireland

**Keywords:** inflammation, macrophages, NADPH oxidase, NOX2, LPS, glycolysis, pentose phosphate pathway, reactive oxygen species

## Abstract

Macrophages undergo a metabolic switch from oxidative phosphorylation to glycolysis when exposed to gram-negative bacterial lipopolysaccharide (LPS), which modulates antibacterial host defence mechanisms. Here, we show that LPS treatment of macrophages increased the classical oxidative burst response via the NADPH oxidase (NOX) 2 enzyme, which was blocked by 2-deoxyglucose (2-DG) inhibition of glycolysis. The inhibition of the pentose phosphate pathway with 6-aminonicotinamide (6-AN) also suppressed the LPS-induced increase in NOX2 activity and was associated with a significant reduction in the mRNA expression of NOX2 and its organizer protein p47phox. Notably, the LPS-dependent enhancement in NOX2 oxidase activity was independent of both succinate and mitochondrial reactive oxygen species (ROS) production. LPS also increased type I IFN-β expression, which was suppressed by 2-DG and 6-AN and, therefore, is dependent on glycolysis and the pentose phosphate pathway. The type I IFN-β response to LPS was also inhibited by apocynin pre-treatment, suggesting that NOX2-derived ROS promotes the TLR4-induced response to LPS. Moreover, recombinant IFN-β increased NOX2 oxidase-dependent ROS production, as well as NOX2 and p47phox expression. Our findings identify a previously undescribed molecular mechanism where both glycolysis and the pentose phosphate pathway are required to promote LPS-induced inflammation in macrophages.

## 1. Introduction

Host responses to infection usually result in macrophage activation leading to the release of pro-inflammatory mediators, which are thought to be linked to the upregulation or downregulation of specific metabolic pathways [1]. For example, during an immune response, the upregulation of glycolysis is critical for the rapid production of ATP, the production of multiple intermediates and cofactors, such as NADH, as well as the production of ribose, fatty acids and amino acids [2]. Through breaks in the tricarboxylic acid (TCA) cycle and the accumulation of the metabolite succinate, HIF1-α is stabilised, and this can promote the upregulation of genes involved in glycolysis and also interleukin (IL)-1β [3,4]. Succinate accumulation can also drive reverse electron transfer, increasing electron leakage in the mitochondria, thus leading to the production of mitochondrial reactive oxygen species (ROS) [5]. Consequently, the M1-activated macrophage drives glycolysis, which ultimately leads to inflammation, but the details of the molecular pathway involved are yet to be fully defined.

Due to the rapid rates of ROS production and the potential indiscriminate toxic nature of these molecules, ROS are subcellularly compartmentalised, which is particularly important in macrophages and neutrophils where ROS can be generated in very high amounts. For example, NOX2-derived phagosomal ROS provide resistance and aid in the phagocytosis and killing of pathogenic bacteria and this requires high-level ROS production [6]. The importance of this antimicrobial effect is illustrated in patients with chronic granulomatous disease (CGD) who have an inability to generate sufficient ROS within phagosomes, due to defective NOX isozymes [7]. Excessive NOX2 activation, on the other hand, has been implicated in multiple inflammatory diseases including cardiovascular disease, chronic obstructive pulmonary disease, cancer and even in infectious diseases such as influenza [8,9,10]. The mechanism of NOX2 activation by Toll-like receptors (TLR) remains largely unknown; however, it at least partially involves protein kinase C signalling pathways, facilitating assembly, translocation and subsequent activation by phosphorylation of the NOX2 subunits [11,12,13]. Controlling NOX2 oxidase production of ROS is, therefore, essential to limit inflammatory disease processes, but equally important in the reverse case to drive localised ROS production in response to phagocytosed bacterial pathogens.

LPS induces a potent inflammatory response by activation of the cell surface receptor TLR4, which activates specific cytokine signalling pathways but also involves metabolic reprogramming, including the upregulation of glycolysis and the pentose phosphate pathway (PPP). This affords at least two benefits to an activated macrophage; it allows for rapid generation of energy products such as ATP to support proliferation, as well as generating metabolic intermediates that contribute to a pro-inflammatory phenotype [2]. For example, the glycolytic enzyme hexokinase 1 has been shown to promote the NLRP3 inflammasome complex, regulating IL-1β through caspase-1 induction [14]. The PPP also promotes inflammation by generating nucleotides, amino acid synthesis and ribose production, all of which aid the reprogramming of inflammatory gene expression. Another product of the PPP is NADPH, an important cofactor in lipid and nucleic acid synthesis. NADPH is involved in the generation of the antioxidant glutathione (GSH), which acts a powerful ROS scavenger, particularly within the mitochondria. However, NADPH can also serve to fuel ROS generation through the NOX NADPH oxidase enzyme family including the prototypical NOX2 oxidase, thus also acting as a pro-oxidant.

A key component of an inflammatory response to an infection is the early release of type I interferons (IFNs), including IFN-α and IFN-β, which use interferon stimulatory genes (ISGs) to modulate the cell cycle which are used to suppress infection, to upregulate antigen presentation in innate immune cells and to activate T and B cells [15]. IFNs are generally produced upon activation of TLRs including TLRs 3, 4, 7 and 9 by double-stranded RNA, LPS, single-stranded RNA and double-stranded DNA, respectively. IFN-β has also been shown to upregulate cytochrome B beta chain (CYBB) expression, the gene responsible for the transcription of the NOX2 subunit [16]. Interestingly, NOX2-derived H_2_O_2_ negatively regulates TLR7 through oxidation of key cysteine residues, inhibiting the production of IFN-β [17]. Others have shown IFN-β as not only mediating a virus-induced increase in glucose uptake, indicating an increase in glycolysis, but pharmacological inhibition using 2-deoxy-d-glucose (2DG) significantly suppressing IFN-β protein production [18]. We therefore hypothesised that IFN-β plays a key role in the regulation of NOX2-derived ROS generation, which could be modulated by a glycolytic switch. In the present study, we show that IFN-β, alongside NADPH generation, is a key regulator of the NOX2-dependent oxidative burst in macrophages, and we implicate glycolysis as the key upstream process of NOX2-derived ROS, with glycolysis also being a significant regulator of IFN-β, likely by the PI3K/Akt pathway [18]. Thus, this positive feedback loop between NOX2-derived ROS and IFN-β, driven by LPS and thereby TLR4, has implications in the control of metabolic switching during an immune response, as well as the prevention of excessive inflammation and for cancer growth/progression.

## 2. Methods

### 2.1. Chemicals

LPS (Invivogen, Thermo Fisher Scientific, Carlsbad, CA, USA) and 2-DG (Sigma-Aldrich, St. Louis, MO, USA, Cat no. D8375) were dissolved in H_2_O at a concentration of 1 mg/mL and 1 M, respectively. 6-aminonicotinamide (6-AN; Sigma-Aldrich, St. Louis, MO, USA, Cat no. A68203) was dissolved in dimethyl sulfoxide (DMSO; Sigma-Aldrich, St Louis, MO, USA, cat. no. 472301; 100%) at a concentration of 200 mM. L-012 (WAKO Chemicals, Richmond, MO, USA, cat. no. 120-04891) and phorbol 12,13-dibutyrate (PDB; Sigma-Aldrich, St Louis, MO, USA, cat. no. P1269-5MG) were dissolved in DMSO in 10 μL and 5 μL aliquots, respectively, at a concentration of 1 × 10^−2^ M. IFN antibody receptor 1 (IFNAR; Bio X cell, Lebanon, NH, USA, Cat no BE0241) was diluted 1:1000 in cell culture medium. Apocynin (Sigma-Aldrich, St. Louis, MO, USA, Cat no. A10809) was dissolved in DMSO at a concentration of 300 mM. Chemicals were stored at −20 °C and rapidly thawed when required, apart from 6-AN and apocynin, which were freshly prepared before each use.

### 2.2. Cell Culture and Maintenance

The immortalised mouse macrophage cell line RAW 264.7 was maintained in Dulbecco’s Modified Eagle’s Media (DMEM; Langley, OK, Gibco, USA), supplemented with 4.5 g/L of glucose, 110 mg/L of sodium pyruvate, 10% (*v*/*v*) fetal bovine serum (FBS; St. Louis, MO, Sigma-Aldrich, USA) and 1% (*v*/*v*) penicillin/streptomycin (Langley, OK, Gibco, USA), and kept at 37 °C with a humidified mixture of 5% CO_2_ and 95% O_2_. Cells were sub-cultured via scraping when confluency reached 80–90% and seeded into a 96-well plate (40,000 cells/well), a 6-well plate (700,000 cells/well) or into T25 flasks (10^6^ cells/flask).

### 2.3. Examination of ROS via L-012 Chemiluminescence

A total of 40,000 cells were seeded overnight in triplicates into a 96-well plate (opti-view), with complete DMEM medium added to a total of 200 µL per well. Oxidative burst was then measured as previously described. Briefly, cells were washed in pre-warmed Krebs-HEPES buffer, then treated with Krebs-HEPES consisting of L-012 (1 × 10^4^ M) and PDB (1 × 10^6^ M) into each well, including blank wells, to account for background luminescence, and immediately run on the CLARIOstar (BMG Labtech, Ortenberg, Germany) under the following conditions: 11 mm focal height, 1 multichromatic, no filter emission, top optic, 1 kinetic window, for 60 cycles with an interval time of 1 s and a cycle time of 100 s. The data are represented as the average of the 60 cycles. Individual cycles were obtained by the average luminescence of the triplicates subtracted by the average luminescence of the blanks. The average was calculated as the average readings over the 60 cycles for each sample subtracted by the average readings for the blanks over the 60 samples. The data were then divided by their respective controls to obtain a fold change value.

### 2.4. Examination of Gene Expression Using qPCR

Cold PBS was used to wash the cells. Samples were then lysed with 350 µL of BME/RLT (1:100) and supernatant was then transferred onto clean Eppendorf tubes. The samples were then spun at 10,000 g for 5 min. Total RNA was then extracted using the RNeasy kit (Qiagen) according to manufacturer’s instructions. Briefly, the supernatants were mixed thoroughly with 350 µL of 70% ethanol, and then loaded onto RNeasy spin columns. The columns were spun down at 8000 g for 15 s; cytochrome B beta chain (CYBB) and the flowthrough were discarded. Then, 700 µL of RW1 was added to each sample and centrifuged at 8000× *g* for 15 s with flowthrough discarded. In addition, 38 µL of combined DNAse I and RDD buffer (Qiagen; 1:8 dilution) was added directly to the membrane and incubated for 15 min at room temperature. The spin columns underwent two washes of 500 µL of RPE buffer at 8000 g for 15 s and 2 min, respectively, then were transferred onto a clean collection tube and spun at 10,000 g for 1 min to remove any contaminants. Spin columns were then transferred onto clean Eppendorf tubes, and 30 µL of RNAse-free water was added directly onto the membrane. Samples were incubated at room temperature for 1 min and then centrifuged at 8000× *g* for 1 min, with the spin columns discarded.

A total of 2 µL of the mRNA was then loaded onto the Nanodrop 2000 (Thermo Fisher Scientific, USA) at 260 nm and 280 nm to measure purity and concentration, respectively. Then, 1 μg of RNA was added into a mixture containing 2 μL 10× RT buffer, 0.8 μL dNTP mix (100 nM), 2 μL 10× RT random primers, 1 μL RNase-free water and 1 μL MultiScribe reverse transcriptase (high-capacity DNA RT kit, Applied Biosystems, Thermo Fisher Scientific, USA) and RNAse-free water was added to reach a final volume of 20 μL. The samples with reaction cocktail were then transcribed to cDNA using the Veriti 96-Well Thermal Cycler (Applied Biosystems, Thermo Fisher Scientific, USA) at 25 °C for 10 min, 37 °C for 120 min and 85 °C for 5 min, in that order. The cDNA was stored at −20 °C prior to PCR.

The Taqman Fast Master Mix was used to analyse gene expression of IL-1β, IFN-β, CYBB (NOX2 subunit) and NRF1 (p47phox), as well as the housekeeping gene RPS18. Respective gene primers (Applied Biosystems, Thermo Fisher Scientific, USA) were used to quantify gene expression. Then, 1:5 dilutions of each sample were prepared for each gene (IFN-β kept neat due to low levels of expression), and 1.5 µL of each sample was dispensed in a 384-well plate (Applied Biosystems, Thermo Fisher Scientific, USA) in triplicates. A 6 µL cocktail containing 3.75 µL of the Taqman Fast master mix, 1.875 µL of RNAse-free water and 0.375 µL of the respective gene primers were added to each sample well, centrifuged at 400× *g* for 5 min at 4 °C. Plates were sealed (Life Technologies, Thermo Fisher Scientific), and real-time quantitative polymerase chain reaction (qPCR) was then performed using the QuantStudio 7 Flex (Applied Biosystems, Thermo Fisher Scientific, USA) with the following protocol: 2 min at 50 °C, 2 min at 95 °C, followed by 40 cycles of 1 s at 95 °C and 20 s at 60 °C. All samples were read using the FAM fluorophore. Gene expression was normalised to RPS18 for each sample and recorded as relative to the control groups. Data obtained from the threshold baseline (CT) were measured automatically during the beginning of the rising linear phase. ΔΔCT was calculated as the difference between each ΔCT and the control ΔCT. Fold changes were then calculated as 2^ΔΔCT^.

### 2.5. Quantification of Protein Using ELISAs

IFN-β was quantified on cell supernatants using the Mouse IFN-beta DuoSet ELISA kit (R&D Systems, Minneapolis, MN, USA, cat no. DY-8234-05) according to manufacturer’s instructions. Briefly, plates were coated with 50 µL of the diluted capture antibody and incubated at room temperature overnight. Plates were aspirated and washed 3 times in PBS-T, and then 150 µL of 1% BSA in PBS (*w*/*v*) was added to each well to block for 1 h at room temperature. Plates were aspirated and washed 3 times in PBS-T, and 50 µL of the samples and standards were added to the wells and incubated for 2 h. Samples were washed 3 times with PBS-T, and 50 µL of the working dilution of the streptavidin–HRP was added to each well and incubated in the dark for 20 min. Wells were washed three times with PBS-T and 50 µL of the substrate solution was added to each well and incubated in the dark for 20 min. Then, 25 µL of the stop solution was added to each well and the plate was read on the CLARIOstar at an absorbance wavelength of 450 nm, with values corrected via a reading of 540 nm and subtracted by a blank reading. IFN-β was determined via readings of absorbance against the standard curve.

### 2.6. Statistical Analysis

All statistical tests were performed using GraphPad Prism (GraphPad Software Version 7.0, San Diego, CA, USA). Statistical tests were performed using a one-way ANOVA followed by Tukey’s post hoc test for multiple comparisons unless otherwise stated. *p* < 0.05 was taken to indicate statistical significance. Each *N* number indicates a different passage of cells used or a sample coming from one mouse.

## 3. Results

### 3.1. LPS Promoted a Glycolytic-Dependent NOX2 Oxidase-Dependent Oxidative Burst Response in Macrophages

**Figure 1:**
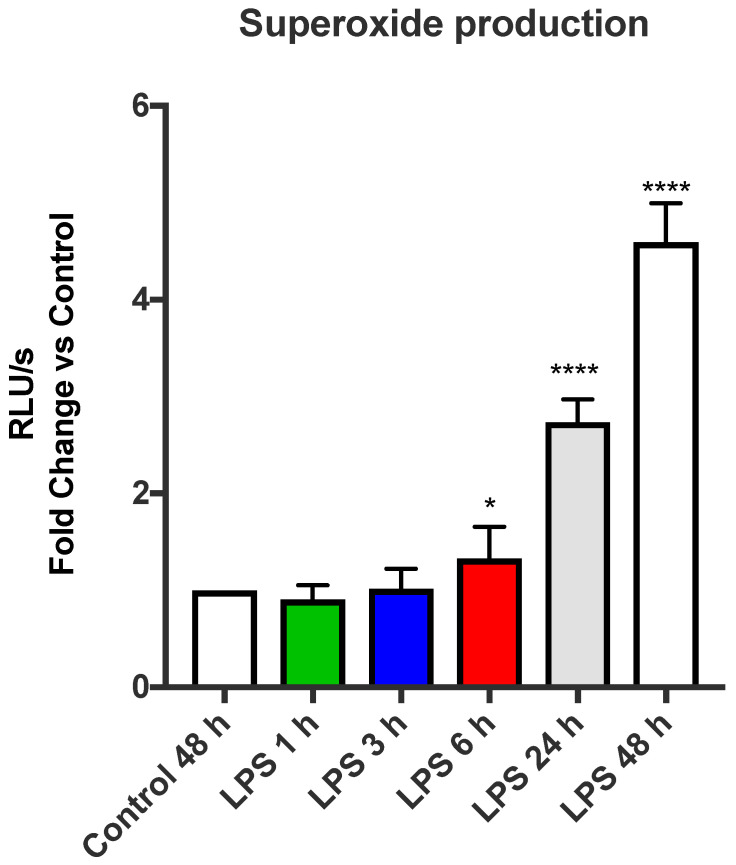
TLR4 activates the NOX2 oxidase-dependent oxidative burst in macrophages. Oxidative burst measured by L-012-enhanced chemiluminescence in RAW 264.7 cells stimulated with the TLR4 agonist LPS (100 ng/mL) over 48 h, 24 h, 6 h, 3 h and 1 h. Data are presented as mean ± SEM of *n* = 7–8 experiments. Data are expressed as a fold change against the respective control in relative light units (RLU), measured as an average of triplicates over a 60-cycle period, subtracted by blank readings. Statistical analysis was conducted via a Student’s *t*-test. * indicates *p* < 0.05 against control and **** indicates *p* < 0.0001 against control.

TLR4 activation drives glycolytic-dependent inflammation and mitochondrial ROS generation in response to LPS stimulation [4,19]. We employed the L-012-enhanced chemiluminescence assay, which we have previously shown to have specific activity for NOX2 [17], to measure ROS production. To elucidate the timing of this oxidative burst response, a time-course series was constructed at 1, 3, 6, 24 and 48 h treatments of LPS (Figure 1). A small ~1.3-fold increase was observed at the 6 h timepoint following LPS treatment, which was further enhanced at 24 h with a ~2.8-fold and ~4.6-fold increase at the 48 h post-LPS treatment, relative to the control (Figure 1). No significant alterations in ROS generation were observed at the 3 h or the 1 h timepoints.

### 3.2. Mitochondrial Activation Did Not Induce a NOX2 Response

**Figure 2:**
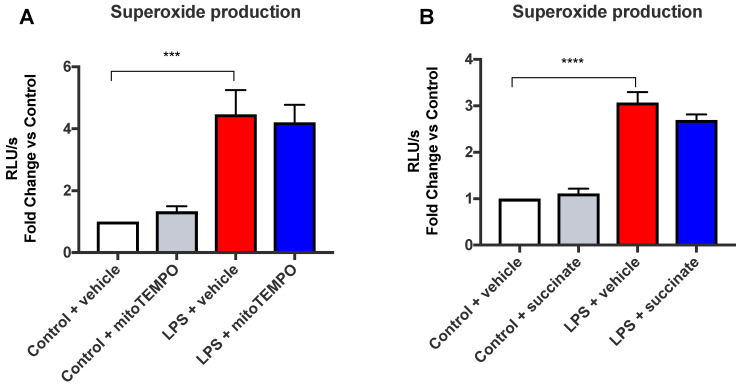
Mitochondrial activity has no effect on the NOX2 oxidase-dependent oxidative burst in macrophages. Oxidative burst measured by L-012-enhanced chemiluminescence in RAW 264.7 cells pre-treated with (**A**) MitoTEMPO (1 mM) for 1 h, or (**B**) succinate (5 mM) for 3 h, followed by LPS (100 ng/mL) stimulation for 24 h. Data are presented as mean ± SEM of *n* = 8–9 experiments. Data are expressed as a fold change against the respective control in relative light units (RLUs), measured as an average of triplicates over a 60-cycle period, subtracted by blank readings. Statistical analysis was conducted via a one-way ANOVA followed by Tukey’s post hoc test for multiple comparisons. *** indicates *p* < 0.001 and **** indicates *p* < 0.0001 against control.

Direct communication between the mitochondria and NOX2 has been reported as a reason for increases in the oxidative burst and may involve a positive feedback mechanism [20]. We therefore examined whether mitochondrial activation and ROS generation were the primary mechanisms for the enhanced oxidative burst in response to the TLR4 ligand LPS. Succinate is a metabolite involved in the TCA cycle, and it is well-established that during inflammation, a build-up of succinate causes upregulation of pro-inflammatory gene expression as well as mitochondrial ROS generation [4,21]. In both untreated and cells treated with LPS, diethyl succinate (cell-permeable succinate which will henceforth be known as succinate) failed to increase the oxidative burst response (Figure 2). To further confirm that mitochondrial ROS was not the stimulus for an enhanced oxidative burst, MitoTEMPO, a targeted mitochondrial ROS scavenger, was added to LPS-treated cells, but MitoTEMPO failed to significantly alter LPS-oxidative burst (Figure 2). Taken together, our findings suggest that mitochondrial ROS does not influence NOX2 oxidase in macrophages, with glycolysis being an upstream regulator independently driving both mitochondrial- and NOX2-derived ROS.

### 3.3. Glycolysis Modulates Type I IFN and NOX2 Gene Expression

**Figure 3:**
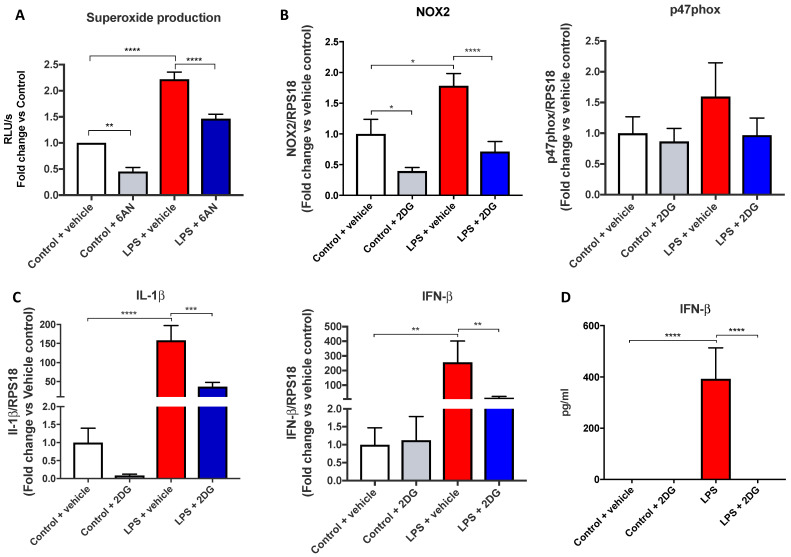
Glycolysis modulates NOX2-dependent ROS generation and IFN-β production. (**A**) Oxidative burst measured by L-012-enhanced chemiluminescence in RAW 264.7 macrophages, (**B**,**C**) mRNA expression measured using qPCR, or (**D**) IFN-β protein measured by ELISA. Cells were pre-treated with 2-DG (1 mM) for 3 h before being stimulated by LPS (100 ng/mL) for 24 h. Data are presented as mean ± SEM of *n* = 8–9 experiments. Data are expressed as (**A**) a fold change against the respective control in relative light units (RLU), measured as an average of triplicates over a 60-cycle period, subtracted by blank readings or the fold change against the vehicle control of a peak response over an average of triplicates over the 60-cycle period, subtracted by blank readings, (**B**,**C**) fold change vs. control against the housekeeping gene RPS18, or (**D**) in pg/mL. Statistical analysis was conducted via a one-way ANOVA followed by Tukey’s post hoc test for multiple comparisons. * indicates *p* < 0.05, ** indicates *p* < 0.01, *** indicates *p* < 0.001 and **** indicates *p* < 0.0001.

We then determined whether the enhancement of the oxidative burst response following LPS occurred in a glycolysis-dependent manner. Pre-treatment with 2-DG resulted in a ~2-fold reduction in the oxidative burst response in the untreated control cells (Figure 3A). Following 2-DG pre-treatment, the LPS-induced enhancement of the oxidative burst displayed a ~1.6-fold reduction. This NOX2 response was associated with a significant ~1.8-fold increase in NOX2 gene expression, which was sensitive to 2-DG, but we observed no changes in p47phox expression (Figure 3B) in response to LPS treatment. Consistent with Tannahill et al., 2013, LPS caused significant increases in IL-1β gene expression, which was markedly suppressed by 2-DG (Figure 3C). We also observed a significant ~250-fold increase in IFN-β expression following LPS stimulation (Figure 3C), and this response was significantly reduced to ~15-fold increase above the vehicle control in the presence of 2-DG. Due to the significant reduction in mRNA expression of IFN-β, we performed ELISAs on the lysates to confirm whether glycolysis inhibits IFN-β protein production. Cells stimulated by LPS produced on average ~400 pg/mL of IFN-β protein compared to unstimulated controls, which produced very little IFN-β (Figure 3D). Further, 2-DG completely inhibited IFN-β protein production, implicating glycolysis as a major regulator of IFN-β mRNA expression and protein production in macrophages, thus suggesting a critical regulatory role.

### 3.4. The PPP Regulates NOX2 Activity and IFN-β Production

**Figure 4:**
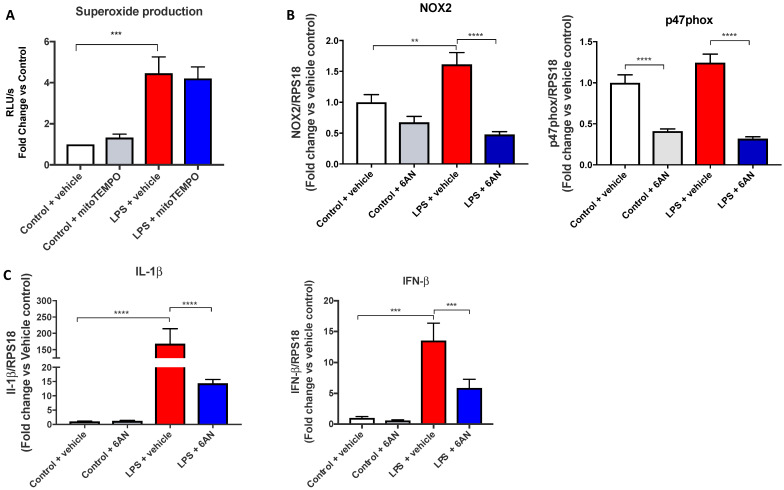
The pentose phosphate pathway is a regulator of NOX2 activity and expression and of inflammation. (**A**) Oxidative burst measured by L-012-enhanced chemiluminescence in RAW 264.7 macrophages, (**B**,**C**) mRNA expression measured using qPCR. Cells were co-treated with 6-AN (200 µM) and LPS (100 ng/mL) for 24 h. Data are presented as mean ± SEM of *n* = 8–9 experiments. Data is expressed as (**A**) a fold change against the respective control in relative light units (RLU), measured as an average of triplicates over a 60-cycle period, subtracted by blank readings or the fold change against the vehicle control of a peak response over an average of triplicates over the 60-cycle period, subtracted by blank readings, or (**B**,**C**) fold change vs. control against the housekeeping gene RPS18. Statistical analysis was conducted via a one-way ANOVA followed by Tukey’s post hoc test for multiple comparisons. ** indicates *p* < 0.01, *** indicates *p* < 0.001 and **** indicates *p* < 0.0001.

As 2-DG inhibition of glycolysis can also inhibit the PPP, we wanted to determine the contribution of the PPP. Using 6-AN, an inhibitor of glucose-6-phosphate dehydrogenase, NOX2-dependent ROS generation was significantly inhibited by ~1.5-fold following LPS treatment (Figure 4A), which was associated with a significant ~3-fold suppression of both NOX2 and p47phox gene expression (Figure 4B). Furthermore, IL-1β and IFN-β expression was significantly suppressed (~10-fold and ~2.5-fold, respectively) with 6-AN following LPS stimulation (Figure 4C). These findings suggest that the PPP is a strong regulator of the inflammatory cytokine response and the NOX2-dependant oxidative burst.

### 3.5. IFN-β Stimulates the Oxidative Burst through Increased NOX2 Expression

**Figure 5:**
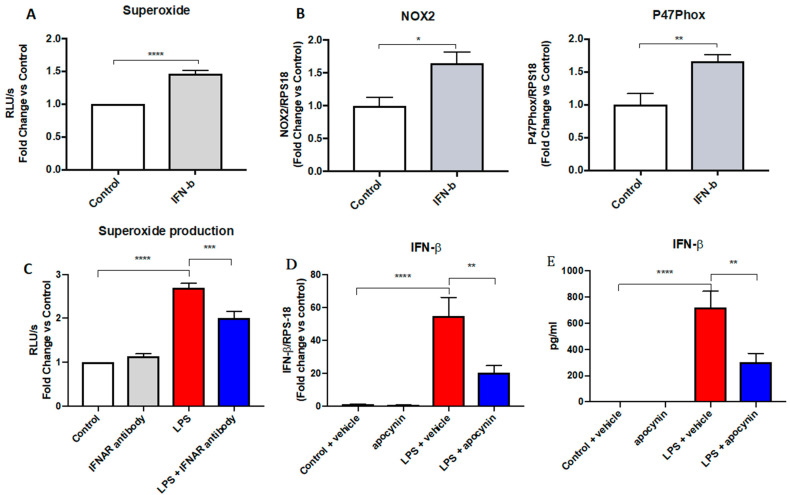
IFN-β is involved in an intricate feedforward loop by increasing the NOX2-dependent oxidative burst. (**A**–**C**) RAW 264.7 cells following 24 h treatment of recombinant IFN-β (100 ng/mL) measuring; (**A**,**C**) oxidative burst measured by L-012-enhanced chemiluminescence, (**B**) mRNA expression measured using qPCR; (**D**,**E**) RAW 264.7 cells pre-treated with apocynin (300 µM) followed by 24 h treatment of LPS (100 ng/mL). Data are presented as mean ± SEM of *n* = 4–8 experiments. Data are expressed as (**A**,**D**) fold change vs. control relative to the housekeeping gene RPS18 and (**B**) relative light units (RLU), measured as an average of triplicates over a 60-cycle period, subtracted by blank readings or the fold change against the vehicle control of a peak response over an average of triplicates over the 60-cycle period, subtracted by blank readings, (**C**) Fold change vs. control relative to the housekeeping protein β-actin, and E) in pg/mL. Statistical analysis was conducted using (**A**–**C**) an unpaired Student’s *t*-test, or (**D**,**E**) a one-way ANOVA followed by Tukey’s post hoc test for multiple comparisons. * indicates *p* < 0.05, ** indicates *p* < 0.01, *** indicates *p* < 0.001 and **** indicates *p* < 0.0001.

Due to the pronounced inhibition of IFN-β expression because of both glycolytic and PPP inhibition following LPS macrophage activation, we explored the specific effects of IFN-β on the NOX2 complex and the associated oxidative burst. Recombinant IFN-β caused a significant ~1.5-fold increase in the NOX2 oxidase response (Figure 5A). This increase in ROS generation was associated with a ~1.5-fold increase in the mRNA expression of NOX2 and p47phox (Figure 5B). As supporting evidence for a pro-oxidant effect of IFN-β, blockage of the IFNAR receptor reduced the maximal oxidative burst capacity in LPS-stimulated cells by ~25% (Figure 5C). Taken together, this suggests that IFN-β can modulate NOX2-derived ROS generation.

Due to the negative feedback loop involving TLR7 and NOX2 [17], we proceeded to determine whether a similar phenomenon occurs with TLR4 to reduce the efficacy of the type I IFN response. To address this, we made use of apocynin, an inhibitor of the phagocytic NOX2 oxidase that also possesses the scavenging activity of ROS. Pre-treating cells with apocynin decreased the mRNA expression of IL-1β by ~3 fold, signifying a lower activation state in these cells. Similarly, a ~3-fold decrease in the IFN-β mRNA expression in LPS-stimulated cells compared to the respective control was observed (Figure 5D). Confirming these results, apocynin inhibited IFN-β protein expression by ~2-fold (Figure 5E), thus demonstrating the positive feedback relationship between IFN-β and NOX2 activity.

## 4. Discussion

Although ROS play important roles in physiological redox signalling, oxidative stress due to excessive ROS is detrimental during an acute innate immune response. Indeed, ROS have been implicated in many acute infectious and inflammatory diseases caused by viruses and bacteria [17,22,23]. Therefore, fine-tuning ROS production, and containing ROS within specific subcellular compartments, may limit excessive collateral damage in a range of inflammatory diseases. In this study, we described for the first time that glycolysis acts as a central regulator of NOX2-derived ROS in macrophages. We showed that glycolysis increases the expression of NOX2 and p47phox to increase the capacity of macrophages to produce ROS. Intriguingly, our data and previous findings suggest that the induction of the glycolytic switch results in an increase in NADPH levels, which serves as an electron donor for NOX2 activity. Glycolysis results in an increase in IFN-β, which we showed causes a transcriptional upregulation of NOX2 and p47phox. These findings led us to propose a novel metabolic-dependent feedback loop between NOX2-derived ROS and type I IFN.

LPS is a powerful inducer of inflammation in a glycolytic-dependent manner [4]. We wanted to determine whether LPS was able to induce NOX2-derived ROS generation in a glycolytic-dependent manner. LPS was capable of enhancing the oxidative burst response in a glycolytic-dependent process in macrophages, providing evidence that a switch to glycolysis allows macrophages to have a heightened capacity to generate ROS via NOX2 oxidase. Glycolysis influences mitochondrial function [24] and, indeed, upregulation of the glycolytic pathway causes breaks in the tricarboxylic acid cycle, including one immediately following the metabolite succinate [4]. Succinate does one of two things: it stabilises HIF1-α, which promotes the upregulation of IL-1β inflammatory gene expression, as well as multiple genes involved in glycolysis [4,25]. It also drives succinate dehydrogenase in oxidative phosphorylation, to drive mtROS through reverse electron transfer [21,26]. Driving reverse electron transfer through succinate accumulation has been shown to drive a strong mtROS response [4]. Indeed, diethyl succinate not only stimulates mtROS, but also stimulates the pro-inflammatory cytokine IL-1β [4,21]. As such, we used diethyl succinate to assess whether succinate is involved in the LPS-dependent induction of the NOX2 oxidase. Our data showed that succinate pre-treatment had no effect on NOX2 oxidase activity in macrophages. To further support this notion, we found that MitoTEMPO, the targeted mitochondrial ROS scavenger, also had no effect on the NOX2 oxidase-dependent oxidative burst. Therefore, these findings suggest that succinate production driven by glycolysis does not alter ROS production by NOX2 oxidase, but rather has powerful modulatory effects on mtROS production. They also suggest that an additional glycolytic-dependent process modifies NOX2 activity in response to LPS. A strong candidate for such a mechanism could be attributed to the activities of the PPP, and in this regard, glycolysis also has inputs into the PPP, i.e., glucose-6-phosphate generation via glycolysis feeds into the PPP. We therefore wanted to determine whether the PPP contributes to the LPS-dependent enhancement of the NOX2 oxidative burst. Using 6-AN, an inhibitor of PPP, we observed a significant reduction in the expression of NOX2 and p47phox, as well as a reduction in the oxidative burst with a similar efficacy to 2-DG; therefore, it is likely that glycolysis regulates the PPP to enhance NOX2 activity. We concluded that glycolysis and PPP drive NOX2 oxidase ROS production in response to LPS and that this is completely independent of mitochondrial ROS and, therefore, most likely only involves TLR4-induced endosomal NOX2 oxidase ROS production.

The LPS-dependent enhancement in NOX2 oxidase activity appeared to be promoted by the PPP, and this energy pathway has two important outcomes: the production of nucleotides and NADPH. NADPH is an electron donor that possesses multiple functions including its use by oxidases to fuel superoxide production, and paradoxically, to generate glutathione, a powerful antioxidant. During infection, it is touted that macrophages use both these functions of NADPH; the first is for ROS production to occur within phagosomes, to clear invading pathogens, and the second to induce an antioxidant program to prevent tissue damage. The findings of the present study suggest that NADPH promotes subcellular-specific oxidant and antioxidant effects. For instance, the NADPH generated promotes phagosomal and endosomal NOX2 activity, by providing electron flow through the NOX2 catalytic subunit. These observations are consistent with the notion that ROS possess specific, subcellular, biological functions and is consistent with antioxidants such as glutathione (primarily located within the cytosol and mitochondria), possessing mitochondrial-specific antioxidant properties.

The present study shows that both glycolysis and the PPP regulate NOX2 oxidase expression and activity to influence the oxidative burst capacity of macrophages. In our study, we focused on IFN-β, a type I IFN that induces an antiviral-like state, which has been previously shown to upregulate the expression of the NOX2 subunit [16,27]. Therefore, we examined the expression of this gene and p47phox, and as housekeeping, we assessed other inflammatory genes well-known to be promoted by glycolysis, such as the pro-inflammatory cytokine IL-1β [4]. We observed that 2-DG treatment of macrophages to inhibit glycolysis markedly suppressed NOX2 and p47phox expression. 2-DG also markedly suppressed IFN-β gene expression and, importantly and consistently with Tannahill et al. (2013), suppressed IL-1β, which are prerequisites of innate immune stimulation and inflammation. Importantly, 2-DG also resulted in a complete suppression in IFN-β protein production. We therefore surmised that glycolysis promotes IFN-β expression as well as NOX2 expression to modulate the oxidative burst response. When we explored this further using the PPP inhibitor 6-AN, we observed a significant decrease in the expression of NOX2 and p47phox, as well as IFN-β and IL-1β. We also demonstrated that exogenous administration of recombinant IFN-β to macrophages resulted in an increase in NOX2 and p47phox expression as well as an increase in ROS production. Therefore, it appears that glycolysis and PPP induction increase IFN-β expression, which drives NOX2 oxidase expression and together with the PPP, drives NADPH, the major electron donor for NOX2, providing a dual mechanism for NOX2 to substantiate an oxidative burst response in macrophages.

Whilst our study employs a well-established cell culture model system for investigating LPS-dependent reprogramming effects in macrophages by use of the RAW 264.7 cell line, it does have the limitation of it not employing primary cells. It is important to note that the bioenergetics of cells vary significantly (Kramer et al., 2014) [28]. For example, monocytes, macrophages, platelets, lymphocytes and neutrophils exhibit unique bioenergetics and their dependence on glycolysis versus oxidative phosphorylation vary significantly. Therefore, how these cells respond to LPS will vary and this certainly warrants future studies that address this via a systematic and comprehensive analysis using specific primary cells.

**Figure 6:**
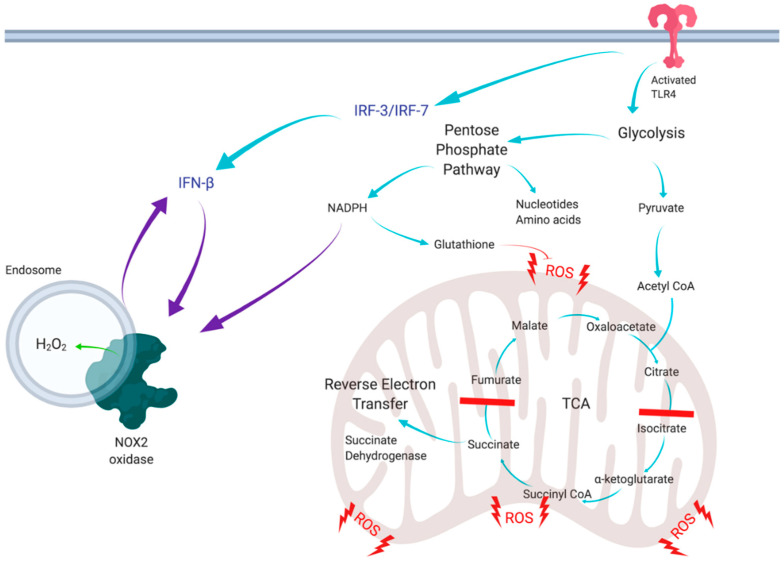
Schematic of mechanisms of the metabolic switch in response to macrophage activation by LPS. Activation induces the metabolic switch, which upregulates glycolysis and the PPP, breaking the TCA cycle and causing a switch from oxidative phosphorylation to reverse electron transfer to generate mitochondrial ROS. Upregulation of glycolysis and the PPP causes release of IFN-β, which is involved in an intricate feedback loop, via increased activation of NOX2 oxidase. NOX2 oxidase, in turn, can increase the production of IFN-β. Blue arrows represent the current literature, and purple arrows represent the novel concepts of this study, including the positive feedback loop. Created with BioRender.com (accessed on 21 March 2022).

In conclusion, the present study has unravelled a novel positive feedforward mechanism between NOX2 oxidase and type I IFN, which is likely to modulate inflammation due to infectious agents (Figure 6). Glycolytic control of NOX2 has multiple implications in immunity and cancer, and involves a control mechanism for ROS production and cytokine signalling. In addition, IFN-β promoting the NOX2 oxidative burst has potential implications in multiple infectious and inflammatory diseases induced by bacterial and viral infections, cancer and cardiovascular disease. Thus, this study provides additional rationale to the emerging concept of glycolytic switch and PPP modulation as a novel therapeutic intervention strategy for inflammatory diseases.

## Data Availability

Data is contained within the article.
